# Recreational Physical Activity as an Independent Predictor of Multivariable Cardiovascular Disease Risk

**DOI:** 10.1371/journal.pone.0083435

**Published:** 2013-12-26

**Authors:** Satvinder S. Dhaliwal, Timothy A. Welborn, Peter A. Howat

**Affiliations:** 1 School of Public Health, Curtin University, Perth, Western Australia, Australia; 2 Department of Endocrinology, Sir Charles Gairdner Hospital, Perth, Western Australia, Australia; 3 Centre for Behavioural Research in Cancer Control, Curtin University, Perth, Western Australia, Australia; INSERM/UMR 1048, France

## Abstract

The role of physical activity in preventing CVD has been highlighted by Professor Jerry Morris in the 1950’s. We report outcome of a 15-year prospective study with the aim to identify whether physical activity showed cardiovascular benefit independent of common risk factors and of central obesity. Baseline data of 8662 subjects, with no previous history of heart disease, diabetes or stroke, were obtained from an age- and gender- stratified sample of adults in Australian capital cities and were linked with the National Death Index to determine the causes of death of 610 subjects who had died to 31 December 2004. The study consisted of 4175 males (age 42.3±13.1 years) and 4487 females (age 42.8±13.2 years). Fasting serum lipid levels, systolic and diastolic blood pressure and smoking habits at baseline were recorded. The Framingham Risk Scores of 15-year mortality due to CHD and CVD were calculated using established equations. Subjects were also asked if they engaged in vigorous exercise, less vigorous exercise or walk for recreation and exercise in the past 2 weeks. Subjects in the high recreational physical activity category were 0.16 (0.06–0.43; *p*<0.001) and 0.12 (0.03–0.48; *p* = 0.003) times as likely as subjects in the low category for CVD and CHD mortality respectively. After adjusting for both the Framingham Risk Score and central obesity (Waist circumference to Hip circumference Ratio), those in the high recreational physical activity group were 0.35 (0.13–0.98) times less likely compared to the low category for CVD mortality. Recreational physical activity independently predicted reduced cardiovascular mortality over fifteen years. A public health focus on increased physical activity and preventing obesity is required to reduce the risk of CVD and CHD.

## Introduction

The role of physical activity in preventing cardiovascular disease (CVD) has been highlighted by Professor Jerry Morris in the 1950’s, where he reported markedly different cardiac death rates in the active conductors of London’s double decker buses compared to their companion bus drivers who are sedentary [1]. He showed that the bus drivers had significantly larger belt sizes, but was unable to distinguish the independent roles of central obesity (waist circumference) versus physical activity. Professor Morris went on to show in a large sample of the postal service that exercise in the course of work was highly protective in a dose-response manner. Also in executives with little opportunity to be active in working hours, those reporting vigorous recreational physical activity had lower rates of CVD [2]. Many observational studies have since confirmed the hypothesis that physical activity promotes cardiovascular health. But after the landmark Framingham studies [3] which reported strong associations between elevated cholesterol levels and hypertension with coronary heart disease (CHD), the epidemiology of cardiovascular disease has consisted largely of measuring modifiable risk factors for which drug therapy is the primary intervention.

Here we report the outcome of a 15-year prospective observation of a population sample whose baseline measures were the conventional cardiovascular disease risk factors, with definitive measures of obesity plus a questionnaire of physical activity at work, home and in leisure. Our aim was to identify whether recreational physical activity showed cardiovascular benefit independent of common risk factors and of central obesity.

## Methods

### Ethics Statement

Ethical clearance for the National Heart Foundation (NHF) 1989 survey was provided by the Australian Institute of Health Interim Ethics Committee, after consultation with the Commonwealth Privacy Commissioner. Participation was entirely voluntary. Those who participated signed an informed consent form attached to the front page of the questionnaire, stating the conditions of participation [4]. The linkage and analysis of the NHF survey data with the National Death Index were approved by the current Ethics Committee of the Australian Institute of Health and Welfare, and complies with the Declaration of Helsinki.

Baseline data of 8662 subjects, with no previous history of heart disease, diabetes or stroke, were obtained from an age- and gender- stratified sample of adults in Australian capital cities [4] and were linked with the National Death Index to determine the causes of death of 610 subjects who had died to 31 December 2004. The study sample consisted of 4175 males (age 42.3±13.1 years) and 4487 females (age 42.8±13.2 years). Of the 346 males who died from all causes, 88 were from cardiovascular disease and 64 were from coronary heart disease. For females, 264 died from all causes and it consisted of 38 from cardiovascular disease and 21 from coronary heart disease. Coding for the causes of death was according to the 10th revision of the International Classification of Diseases (ICD-10): Codes I 00.0 to I 99.9 for CVD deaths and codes I 20.0 to I 25.9 for CHD deaths [5]. Our study determined causes of death using death certificate data. In Australia, identifying causes of death using death certificate data has been shown to have both high sensitivity and positive predictive value, being 88.9% and 96% respectively [6].

Fasting serum lipid levels, systolic and diastolic blood pressure and smoking habits at baseline were recorded [7]. The Framingham risk scores (FRS) of 15-year mortality due to CHD and CVD were calculated using the equations in Anderson’s paper [8] which predict the risk of death due to CHD and CVD for subjects free of heart disease, diabetes or stroke, after accounting for age, gender, systolic blood pressure, total cholesterol, high-density lipoprotein cholesterol, and cigarette smoking. Central obesity was assessed by waist circumference to hip circumference ratio (WHR) using standardized methods [9], and was assessed using two observers.

In the baseline survey, subjects were asked about recreation, sport or health-fitness in the past two weeks. They indicated if they engaged in vigorous exercise (exercise which made them breath harder or puff or pant), if they engaged in less vigorous exercise not causing them to breath harder or if they walked for recreation or exercise [4]. This included a statement of how many sessions or times in the past two weeks plus an estimate of total time spent. Similar questions on vigorous tasks at work or around the house were asked.

The questions on physical exercise were combined to create a composite recreational physical activity score, ranging from 0 (subjects who did not engage in vigorous exercise, less vigorous exercise and walking in the previous 2 weeks) to 7 (subjects who engaged in all forms of exercise in the previous 2 weeks). Scores 0–3 were classified as being in the low category of the composite variable, 4–5 as being in the medium category and scores from 6–7 were classified in high category (Table 1).

**Table 1:** Scoring and categorisation of composite recreational physical activity variable from subjects’ responses to physical activity questions.*

Recreational physicalactivity category	Engaged in vigorousexercise*	Engaged in lessvigorous exercise*	Walking for recreationor exercise*	Recreational physical activity score
High	Yes	Yes	Yes	7
	Yes	Yes	No	6
Medium	Yes	No	Yes	5
	Yes	No	No	4
Low	No	Yes	Yes	3
	No	Yes	No	2
	No	No	Yes	1
	No	No	No	0

In the previous 2 weeks.

Fifteen year mortality data on cardiovascular disease and coronary heart disease from the NHF cohort was analysed using multivariable logistic regression to assess the contribution of physical activity variables, with and without adjusting for CVD-related covariates. Covariates considered in the analyses include the Framingham risk score variables (age, gender, smoking status, systolic blood pressure and total cholesterol to high density lipoprotein cholesterol ratio) and central obesity as assessed by WHR. The effects of physical exercise, Framingham risk score and central obesity were expressed as odds-ratio and associated 95% confidence intervals for CVD and CHD mortality.

P-values less than 0.05 were considered to be statistically significant, and were two-sided. Data was analysed using IBM SPSS Statistics Version 20.

## Results

Baseline characteristics for the 8662 subjects who were free of baseline history of heart disease, diabetes or stroke in 1989 were presented in Table 2. The sample consisted of 4175 males (age 42.3±13.1 years) and 4487 females (age 42.8±13.2 years). During the 15-year mortality follow-up, there were 126 deaths due to cardiovascular disease and 85 deaths due to coronary heart disease. Framingham predicted risk scores were higher in subjects who died from cardiovascular disease and coronary heart disease as compared to survivors. Survivors also had lower waist to hip ratio and were more physically active as indicated by the individual questions on vigorous exercise, less vigorous exercise and engagement in vigorous tasks in the previous 2 weeks. A higher proportion of survivors were engaged in medium to high categories as determined from the composite recreational physical activity variable.

**Table 2:** Characteristics of the cohort of 8662 subjects without angina, diabetes and stroke at baseline.

	CVD Deaths	CHD Deaths	Survivors	Total Cohort
Count	126	85	8052	8662
Male (%)	69.8	75.3	47.6	48.2
Age (years)	59.8±9.9	59.6±9.6	41.5±12.6	42.6±13.1
Body Mass Index (kg/m^2^)	26.7±4.0	26.8±3.4	25.2±4.2	25.3±4.2
Waist to Hip ratio	0.9±0.1	0.9±0.1	0.8±0.1	0.8±0.1
Current smoker (%)	34.9	40.0	23.9	24.5
Systolic Blood Pressure (mmHg)	142.0±22.0	142.2±20.9	124.1±16.9	125.2±17.7
Diastolic Blood Pressure (mmHg)	86.0±12.4	85.2±11.6	77.9±11.0	78.3±11.1
Total Cholesterol (mmol/L)	6.0±1.1	6.1±1.2	5.5±1.1	5.5±1.1
High Density Lipoprotein (mmol/L)	1.2±0.4	1.2±0.4	1.3±0.4	1.3±0.4
Total Cholesterol to HDL ratio	5.3±1.8	5.5±1.9	4.4±1.5	4.4±1.5
Framingham Predicted Risk for CardiovascularDisease death (%)	15.1±11.3	15.8±11.3	2.9±5.0	3.5±5.9
Framingham Predicted Risk for Coronary HeartDisease death (%)	10.5±8.0	11.2±8.1	1.9±3.8	2.3±4.4
Engaged in vigorous exercise in previous 2 weeks (%)	13.5	12.9	33.4	32.2
Engaged in less vigorous exercise in previous 2 weeks (%)	22.2	20.0	31.8	31.0
Walked for recreation or exercise in previous 2 weeks (%)	58.7	62.4	56.1	55.9
Engaged in vigorous tasks in previous 2 weeks (%)	31.0	34.1	40.5	39.9
Recreational physical activity category				
Low (%)	86.5	87.1	66.6	67.8
Medium (%)	10.3	10.6	17.3	16.8
High (%)	3.2	2.4	16.1	15.4

**Figure 1:**
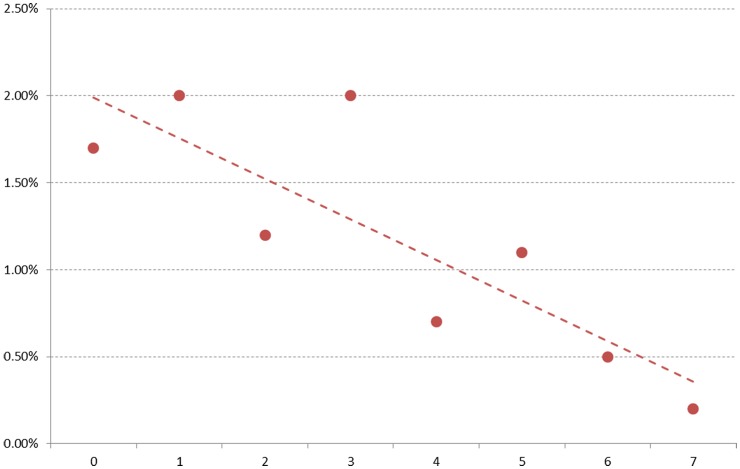
Actual cardiovascular disease mortality by recreational physical activity score.

The association between actual cardiovascular disease mortality and increasing levels of recreational physical activity score were shown in Figure 1. This graph showed a significant negative association between the two variables without adjustment for covariates, indicating lower cardiovascular disease mortality for those subjects who were engaged in higher recreational physical activity.

Table 3 summarised the unadjusted odds-ratio and associated 95% confidence intervals for each physical exercise variable in the baseline survey, and for the composite recreational physical activity categories. Vigorous exercise and less vigorous exercise were associated with a lower risk for cardiovascular and coronary heart disease mortality, without adjustment for covariates. For the composite variable, there was a significant trend towards lower risk of cardiovascular disease and coronary heart disease mortality for the higher categories for recreational physical activity.

**Table 3:** Odds-ratios* and associated 95% confidence intervals for CVD and CHD mortality.

	CVD Deaths*	CHD Deaths*
Vigorous exercise	0.32 (0.19–0.54) *(p<0.001)*	0.31 (0.16–0.59) *(p<0.001)*
Less Vigorous exercise	0.63 (0.42–0.97) *(p = 0.034)*	0.55 (0.33–0.95) *(p = 0.030)*
Walked for exercise	1.12 (0.79–1.61) *(p = 0.520)*	1.31 (0.84–2.04) *(p = 0.230)*
Vigorous tasks	0.67 (0.46–0.98) *(p = 0.040)*	0.78 (0.50–1.22) *(p = 0.275)*
Recreational physical activity category:		
Medium vs. Low	0.48 (0.27–0.85) *(p = 0.012)*	0.49 (0.24–0.98) *(p = 0.043)*
High vs. Low	0.16 (0.06–0.43) *(p<0.001)*	0.12 (0.03–0.48) *(p = 0.003)*

Unadjusted analyses. Statistically significant (p<0.05) odds-ratio of less than 1 suggests protective effect.

The characteristics of the cohort of 8662 subjects were also presented by the Low, Medium and High recreational physical activity categories in Table 4. Subjects in high and medium recreational physical activity categories had lower values on variables associated with cardiovascular and coronary heart disease risk, and with the respective Framingham risk score compared to subjects in the low recreational physical activity category.

**Table 4:** Characteristics of subjects in each of the 3 recreational physical activity categories.

	Recreational physical activity categories		
	*Low*	*Medium*	*High*
Count	5870	1454	1331
CVD death (%)	1.9	0.9	0.3
CHD death (%)	1.3	0.6	0.2
Male (%)	45.0	54.5	55.4
Age (years)	45.5±12.9	37.0±11.5	35.9±11.1
Body Mass Index (kg/m^2^)	25.6±4.5	24.8±3.6	24.4±3.4
Waist to Hip ratio	0.83±0.10	0.82±0.09	0.81±0.08
Current smoker (%)	26.0	22.9	19.5
Systolic Blood Pressure (mmHg)	126.7±18.6	122.4±15.5	121.4±14.4
Diastolic Blood Pressure (mmHg)	79.1±11.2	76.8±10.8	76.1±10.7
Total Cholesterol (mmol/L)	5.6±1.1	5.3±1.0	5.2±1.0
High Density Lipoprotein (mmol/L)	1.3±0.4	1.3±0.4	1.4±0.4
Total Cholesterol to HDL ratio	4.5±1.6	4.2±1.4	4.1±1.4
Framingham Predicted Risk for Cardiovascular Disease death (%)	4.3±6.6	1.9±3.7	1.6±3.1
Framingham Predicted Risk for Coronary Heart Disease death (%)	2.9±4.9	1.1±2.8	0.9±2.4
Engaged in vigorous exercise in previous 2 weeks (%)	0.0	100.0	100.0
Engaged in less vigorous exercise in previous 2 weeks (%)	23.0	0.0	100.0
Walked for recreation or exercise in previous 2 weeks (%)	54.0	50.3	70.4
Engaged in vigorous tasks in previous 2 weeks (%)	36.7	42.9	50.4

The effects of recreational physical activity on CVD and CHD mortality was presented as odds-ratio and associated 95% confidence intervals, with and without adjustment for Framingham risk score (which was computed from age, gender, smoking status, systolic blood pressure and total cholesterol to high density lipoprotein cholesterol ratio), and with adjustment for central obesity (WHR) (Table 5). Without adjustment, subjects in the medium recreational physical activity category were about half as likely as subjects in the low exercise category for CVD and CHD mortality (*p*<0.05). Subjects in the high recreational physical activity category were 0.16 (0.06–0.43; *p*<0.001) and 0.12 (0.03–0.48; *p* = 0.003) times as likely as subjects in the low recreational physical activity category for CVD and CHD mortality respectively. The independent effects of high recreational physical activity on CVD and CHD mortality were significant (*p*<0.05) even after adjusting for the Framingham risk score. After adjusting for both the Framingham risk score and central obesity (WHR), those in the high recreational physical activity group were 0.35 (0.13–0.98) times less likely compared to the low recreational physical activity for CVD mortality (*p*<0.05). In relation to CHD mortality, the effects of recreational physical activity, high versus low, was not significant (*p* = 0.066), possibly due to the lack of statistical power associated with the lower number of subjects with CHD mortality. The independent effects of high recreational physical activity therefore generally persisted after adjustment for covariates associated with higher risk, Framingham risk score variables and central obesity. This confirms that recreational physical activity is an independent predictor of cardiovascular and coronary heart disease mortality.

**Table 5:** Framingham Risk Score and obesity adjusted odds ratios for CVD and CHD mortality.

	Without adjustment	Adjusted for Framingham risk score	Adjusted for Framingham risk score and central obesity
***CVD Deaths***			
Framingham risk score		3.39 (0–3.96)	2.94 (2.47–3.50)
*(per 10% increase)*		*(p<0.001)*	*(p<0.001)*
Waist to Hip ratio			1.50 (1.18–1.90)
*(per 0.1 increase)*			*(p = 0.001)*
Recreational physical activity categories:			
* Medium vs. Low*	0.48 (0.27–0.85)	0.81 (0.44–1.51)	0.85 (0.45–1.58)
	*(p = 0.012)*	*(p = 0.513)*	*(p = 0.605)*
* High vs. Low*	0.16 (0.06–0.43)	0.33 (0.12–0.91)	0.35 (0.13–0.98)
	*(p<0.001)*	*(p = 0.033)*	*(p = 0.046)*
***CHD Deaths***			
Framingham risk score		5.03 (3.96–6.39)	3.98 (3.02–5.26)
*(per 10% increase)*		*(p<0.001)*	*(p<0.001)*
Waist to Hip ratio			1.60 (1.20–2.15)
*(per 0.1 increase)*			*(p = 0.002)*
Recreational physical activity categories:			
* Medium vs. Low*	0.49 (0.24–0.98)	0.88 (0.43–1.81)	0.94 (0.45–1.92)
	*(p = 0.043)*	*(p = 0.733)*	*(p = 0.856)*
* High vs. Low*	0.12 (0.03–0.48)	0.24 (0.06–0.99)	0.26 (0.06–1.09)
	*(p = 0.003)*	*(p = 0.049)*	*(p = 0.066)*

Predictive effect is presented as odds-ratio and associated 95% confidence intervals, before and after adjustment for covariates.

## Discussion

In this population study, a questionnaire identifying recent recreational physical activity predicted cardiovascular mortality over fifteen years. The questionnaire was designed to achieve recall over a limited time span, and the assumption is that this provides an indication of long-term behaviors. Incidental physical activity was not included. Fitness is known to benefit cardiovascular outcomes, and has been attributed to its influence on intermediate factors such as blood pressure, lipids and obesity [10] but there is little evidence for an independent effect, after adjustment for covariates.

Our data suggests a gradient of risk protection from the low to high levels of recreational physical activity. In this study, recreational physical activity has been shown to be an important significant independent predictor of cardiovascular mortality after adjusting for all other intermediate Framingham risk factors and a measure of central obesity. Although adjustment for covariates weakens the relationship between recreational physical activity and subsequent cardiovascular mortality, the predictive ability remains statistically significant. This process of adjustment for covariates provides a conservative estimate of the effect of recreational physical activity and it emphasizes the importance of the protective effect of physical activity on the reduction of risk of cardiovascular mortality, independent of intermediary factors.

When measures of central obesity were included in the logistic regression, body mass index (BMI) did not contribute to risk, but measures of central obesity and specifically the waist to hip ratio had an independent and highly significant contribution to CVD and CHD deaths. Elsewhere we have shown that waist circumference (WC) and more importantly waist to hip ratio predict cardiovascular disease much more effectively than BMI [11] and can predict total mortality [12]. One other prospective study [13] showed that obesity and physical activity predict cardiovascular risk after ten years in a Finnish population sample. Our study is unique in taking into account the measured risk factors and showing independence from them.

More extensively studied cohorts using repeated and detailed measures of exercise confirm a direct graded benefit for increased physical activity. These include the College Alumni Study [14] and the Nurses’ Health Study [15]. There is incontrovertible evidence for a dose response relationship of physical activity in preventing cardiovascular disease, stroke, and hypertension in a comprehensive systematic review (47 reports) but interaction with other factors and possible confounders was not assessed [16]. Thus in the primary prevention of cardiovascular disease, there is a risk reduction of 33%, but can be more than 50% if objective measures of exercise are reported. The level of evidence grade 2A is based on observational studies with overwhelming data, or randomized controlled trials with important limitations, and supports recommendations that apply to most individuals in most settings. Similarly for stroke, the average risk reduction is 31% but this can be as high as 68% with direct measures of fitness, and for hypertension the average risk reduction is 32%, but can be as high as 63% when maximal cardiovascular performance is evaluated.

A recent comprehensive meta-analysis of cohort studies examining the all-cause mortality in general adult populations across many categories of physical activity has been reported recently [17]. Of 80 studies, the median follow-up was 10 years, with the majority (88%) using a single baseline assessment and half (54%) used a brief questionnaire. No associated risk factors were studied but an incremental reduction in death rates was found with increasing physical activity, and was greater in women than in men. Risk reduction per unit of time increase was largest for vigorous exercise. In the CARDIA study, healthy life style, that includes moderate to vigorous physical activity, in young adults translated to a low cardiovascular disease risk profile in middle age, after 20 years [18].

The promotion of increased physical activity is clearly a powerful vehicle for prevention of cardiovascular disease and premature mortality. Every adult without major disease should benefit from increased physical activity, with the greatest health benefits associated with high levels of exertion. Our study confirms the independent role of recreational physical activity in predicting and reducing cardiovascular deaths, even after the common association with conventional risk factors and obesity has been accounted for. These findings support public health endeavor to promote exercise over and above the treatment of conventional risk factors.

Our study has limitations in that there is only one set of baseline measurements although some important measures were assessed twice, to assess measurement error. Our questionnaire on physical activity is not as useful as an objective measure, especially when comparing medium to low levels of physical activity. The end points of the study, deaths due to coronary heart disease and cardiovascular disease, have been established from death certificates and no incidence data is available. The assessment of recreational physical activity recall was over a limited time span, and we made the assumption that this provides an indication of long-term lifestyle behaviors. Another important limitation of this study is that the effect of physical activity cannot be assessed in combination or interaction with other risk factors, due to insufficient sample size related to cardiovascular disease outcomes for each combination of factors.

## Conclusions

This study supports the concept of a public health focus on increased recreational physical activity and preventing obesity which will have major benefits in reducing the risk of cardiovascular disease and coronary heart disease. This will translate to a reduction in the substantial cost burden of multiple drug therapy in the treatment of hypertension and dyslipidaemia. Such a public health focus could shift preventive policies from drug based interventions to that of behavioral, social and environmental programs.

## References

[1] Morris JN, Heady JA, Raffle PA, Roberts CG, Parks JW (1953) Coronary heart-disease and physical activity of work. Lancet 265: 1111–1120. 13110075 10.1016/s0140-6736(53)91495-0

[2] Morris JN, Clayton DG, Everitt MG, Semmence AM, Burgess EH (1990) Exercise in leisure time: coronary attack and death rates. British Heart Journal 63: 325–334. 2375892 10.1136/hrt.63.6.325 PMC1024515

[3] Dawber TR, Moore FE, Mann GV (1957) Coronary Heart Disease in the Framingham Study. American Journal of Public Health 47: 4–24. 10.2105/ajph.47.4_pt_2.4 PMC1550985 13411327

[4] Australian Risk Factor Prevalence Study Management Committee (1990) Survey No 3 1989. Canberra: National Heart Foundation of Australia and Australia Institute of Health.

[5] World Health Organization (1998) International classification of diseases. Manual of the international statistical classification of diseases, injuries, and causes of death, 10th revision (ICD-10). Geneva: WHO.

[6] Boyle CA, Dobson AJ (1995) The accuracy of hospital records and death certificates for acute myocardial infarction. Aust N Z J Med 25: 316–323. 8540872 10.1111/j.1445-5994.1995.tb01896.x

[7] Welborn TA, Dhaliwal SS, Bennett SA (2003) Waist–hip ratio is the dominant risk factor predicting cardiovascular death in Australia. Med J Aust 179: 580–585. 14636121 10.5694/j.1326-5377.2003.tb05704.x

[8] Anderson KM, Odell PM, Wilson PWF, Kannel WB (1991) Cardiovascular disease risk profiles. American Heart Journal 121: 293–298. 1985385 10.1016/0002-8703(91)90861-b

[9] Dhaliwal SS, Welborn TA (2009) Measurement error and ethnic comparisons of measures of abdominal obesity. Preventive Medicine 49: 148–152. 19589354 10.1016/j.ypmed.2009.06.023

[10] Lopez AD, Mathers CD, Ezzati M, Jamison DT, Murray CJL (2006) Global Burden of Disease and Risk Factors. New York: The World Bank and Oxford University Press. 21250374

[11] Dhaliwal SS, Welborn TA (2009) Central Obesity and Multivariable Cardiovascular Risk as Assessed by the Framingham Prediction Scores. The American Journal of Cardiology 103: 1403–1407. 19427436 10.1016/j.amjcard.2008.12.048

[12] Welborn TA, Dhaliwal SS (2007) Preferred clinical measures of central obesity for predicting mortality. European Journal of Clinical Nutrition 61: 1373–1379. 17299478 10.1038/sj.ejcn.1602656

[13] Hu G, Tuomilehto J, Silventoinen K, Barengo N, Jousilahti P (2004) Joint effects of physical activity, body mass index, waist circumference and waist-to-hip ratio with the risk of cardiovascular disease among middle-aged Finnish men and women. European Heart Journal 25: 2212–2219. 15589638 10.1016/j.ehj.2004.10.020

[14] Sesso HD, Paffenbarger RSJ, Lee IM (1998) Physical Activity and Breast Cancer Risk in the College Alumni Health Study (United States). Cancer Causes & Control 9: 433–439. 9794176 10.1023/a:1008827903302

[15] Hu FB, Manson JE, Stampfer MJ, Colditz G, Liu S, et al (2001) Diet, Lifestyle, and the Risk of Type 2 Diabetes Mellitus in Women. New England Journal of Medicine 345: 790–797. 11556298 10.1056/NEJMoa010492

[16] Warburton D, Charlesworth S, Ivey A, Nettlefold L, Bredin S (2010) A systematic review of the evidence for Canada’s Physical Activity Guidelines for Adults. International Journal of Behavioral Nutrition and Physical Activity 7: 39. 20459783 10.1186/1479-5868-7-39 PMC3583166

[17] Samitz G, Egger M, Zwahlen M (2011) Domains of physical activity and all-cause mortality: systematic review and dose–response meta-analysis of cohort studies. International Journal of Epidemiology. 10.1093/ije/dyr112 22039197

[18] Liu K, Daviglus ML, Loria CM, Colangelo LA, Spring B, et al (2012) Healthy Lifestyle Through Young Adulthood and the Presence of Low Cardiovascular Disease Risk Profile in Middle Age: The Coronary Artery Risk Development in (Young) Adults (CARDIA) Study. Circulation 125: 996–1004. 22291127 10.1161/CIRCULATIONAHA.111.060681 PMC3353808

